# Understanding post-conflict mental health needs and co-producing a community-based mental health intervention for older adults in Colombia: a research protocol

**DOI:** 10.1186/s12913-022-07645-8

**Published:** 2022-02-24

**Authors:** Clarissa Giebel, Maria Isabel Zuluaga, Gabriel Saldarriaga, Ross White, Siobhan Reilly, Erica Montoya, Dawn Allen, Ginger Liu, Yeferson Castaño-Pineda, Mark Gabbay

**Affiliations:** 1 Department of Primary Care & Mental Health, University of Liverpool, Liverpool, UK; 2 NIHR ARC NWC, Liverpool, UK; 3 Public Health Faculty, Universidad de Antioquia, Medellín, Colombia; 4 Centre for Applied Dementia Studies, Faculty of Health Studies, University of Bradford, Bradford, UK; 5 NIHR Applied Research Collaboration Yorkshire & Humber, Leeds, UK

**Keywords:** Mental health, Ageing, Violence, Post-conflict, Low- and middle-income countries

## Abstract

**Background:**

Older adults in Colombia have seen a number of stressful life events – including the Colombian armed conflict, forced misplacement and recently COVID-19. These events likely have had and are having a substantial impact on people’s mental health and well-being, whilst mental health care provision in Colombia is not sufficient and often access is limited and unaffordable. Therefore, the aim of this study is to understand the impact of stressful life events on the mental health of older adults living in Colombia, and co-produce, pilot, and evaluate a community-based mental health intervention in Turbo.

**Methods:**

This 3-year international mixed-methods study comprises of three phases: Phase I will explore the impact of stressful life events on the mental health of older adults living in Colombia, and their mental health needs, via quantitative needs assessments and qualitative interviews and focus groups; Phase II will involve synthesising the findings from Phase I as well as conducting a systematic review and qualitative interviews with experts into implementing mental health interventions in LMICs to co-produce a community-based mental health intervention with older adults and local community group leaders and care providers; Phase III will involve the piloting and evaluation of the mental health intervention via quantitative and qualitative assessments. Co-production and public involvement underpin each element of this project.

**Discussion:**

Appropriate mental health care is as important as physical health care, but this study also looks at how we might integrate these findings into community-level public health initiatives for application both within Colombia and more widely in both LMICs and more developed countries. This study protocol will act as a guide for the development and adaptation of psychosocial mental health interventions in different cultures and contexts.

## Background

Colombia has suffered political violence since the mid-twentieth century notably the Colombian armed conflict, an armed conflict between guerrillas groups and the government [1], and more recently drug cartel related violence after the peace treaty with the FARC guerrilla [2]. These severely impact on its citizens. Colombians, as with other populations across the world are now being affected by the global COVID-19 pandemic. How these events are affecting its residents’ mental health, particularly older adults, is unclear though.

The proportion of Colombian older adults (aged 60 +) is expected to rapidly increase by 2050 from 10 to 20% of its current 45 million population. This rise is increasingly impacting upon Colombian communities, particularly in regions like Urabá in the Department of Antioquia, one of 32 departments/regions, that have suffered throughout their history from inequality and lack of development due to inattention from the state and the constant presence of prolonged political conflict that deepens these structural conditions of vulnerability [3]. Uraba Antioqueno has been one of the territories most affected by the Colombian armed conflict in the last six decades. It is considered one of the regions of the Department of Antioquia with the highest numbers of expulsion of people for reasons related to the armed conflict or violence, though at the same time it is one of the main receiving areas of victims from Choco in the Pacific region and the Atlantic coast, as well as the migratory transit of foreigners from Africa and Latin America who are seeking to reach the United States. According to the National Information Network of the Unit for Attention and Comprehensive Reparation to Victims, as of January 2020, 413,397 inhabitants (78.6% of the total regional population of 525,685) were registered as victims of the armed conflict). In Turbo, the proportion is 64.5% (83,993 victims) [4]. In the region, 9.7% (*n* = 14,425) are classed as older adults, living in urban, rural, peri-urban and dispersed rural areas.

Assessing the mental health of the elderly population is especially important in such territories where populations struggle with relative poverty, lack of opportunity and a violent environment, now compounded by Covid-19. Research into the impacts of the armed conflict and other stressful life events in Colombia appears to have focused on the population at large, without specifically focusing on older adults, who have had more time to be faced by these events. Specifically, Cuartas Ricaurte et al. [5] reported that victims of the armed conflict experienced 1.74 higher odds of having a mental health disorder, with higher prevalence in people from more socio-economically disadvantaged backgrounds. However, people do not need a mental health diagnosis to have been emotionally impacted by these events. Burgess and Fonseca [6] showed the emotional distress and poor levels of mental well-being in internally displaced Colombians, leading to the recommendation of locally informed mental health recovery models and interventions. Whilst there is less literature on older Colombians in particular, Flores et al. [7] reported that sexual abuse and displacement due to armed conflict were associated with higher levels of depression in older adults. This is based on quantitative assessments and purely focuses on depression, yet neglects a focus on other measures of mental health, such as quality of life and anxiety, as well as the qualitative experiences of these stressful life events on older adults’ mental health.

Addressing those unmet needs is an important issue, as Colombia, similar to many other low- and middle-income countries (LMICs), has limited mental health care coverage, making it difficult for people in need to receive adequate care [8–11]. Infrastructure and costs of accessing mental health care provide instrumental barriers, whilst stigma surrounding mental health has been reported to be a personal barrier to accessing suitable care [12].

The aim of this joint 3-year project is to understand how political, global and individual stressful life events affect mental well-being among older adults in Colombia, and how their impact can be reduced by a co-produced community intervention. Our research questions are: *(1) What effects have historic and current interpersonal violence and the COVID-19 pandemic had on the mental health of older adults (aged 60* +*) in the District of Turbo Antioquia in Colombia?; (2) What are the best ways to co-produce, evaluate and deliver a psycho-social mental health intervention for older adults in this environment, implement and evaluate its impact on mental health?* Our approach incorporates the co-design of a community-based psychosocial care strategy to address the situations identified at the family and community levels, in coordination with different key actors in the region. Considering the lack of accessible and available mental health care and support in Colombia and LMICs more broadly [12], this study provides an important step in improving our understanding of the need for mental health support whilst also offering a direct solution by developing a community-based mental health intervention, in line with the World Health Organisation’s mental health Gap Action Programme (mhGAP) to scale up mental health services in LMICs [13]. This study protocol provides an overview of the individual components of this large-scale international research, to enable other teams to have a guide to replicate the development of mental health interventions in their cultural settings.

## Methods/Design

**Fig. 1:**
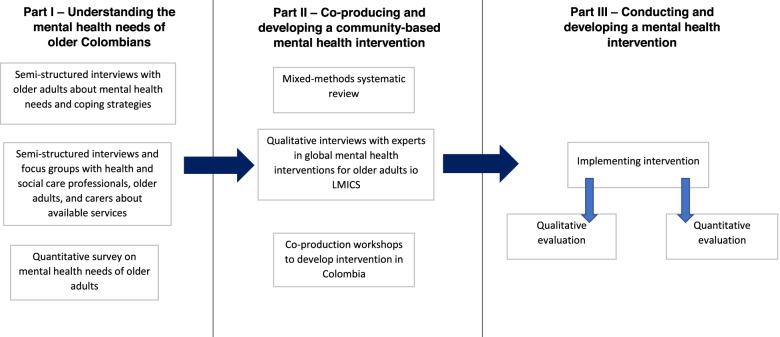
Project overview. Flowchart of the three overarching project components

This 3-year cross-country mixed-methods study comprises of three phases, specifically involving surveys of older-residents’ mental health, wellbeing and functional capacity in contexts of environment, experience of past and current violence, covid-19 additional impacts. This alongside evidence synthesis and expert panels, plus in-depth local co-production will inform the determination of key components of the co-designed intervention, identify existing data and new data required to track the impact of a co-produced community-level psycho-social intervention, in collaboration with existing Municipality health assets and teams, to then be piloted within that community for future robust wider cost effectiveness evaluation and implementation guidance. Figure 1 shows the different elements, and further detail on each phase and study element are provided below.

### Study overview

#### Part I – Understanding the mental health needs of older Colombians

Part I of the project addresses research question 1 and comprises three elements to better understand the impact of stressful life events on the mental health of older Colombians, and that population’s wider mental health needs. Whilst elements are present subsequently, they will be conducted simultaneously. Ethical approval has been obtained for these study elements from the National Faculty of Public Health at the University of Antioquia (Session 244). The first element are 30 semi-structured interviews, conducted either face-to-face or remotely dependant on COVID-19 restrictions, to understand how stressful life-events, including the armed conflict and COVID-19, have impacted on older people’s mental health. The focus is on older people residing in the turbo region. The topic guide will be co-produced with members of the public; focusing on how life experiences are affecting their mental health and general wellbeing, as well as their coping strategies with stressful life events. Transcripts will be translated into English and analysed using thematic analysis^6^ by both Colombian and UK researchers in their respective languages, with regular virtual team analysis meetings including PAs. Constant comparative thematic analysis and interpretation of all qualitative data will be framed within Social Constructivism theory informed by Symbolic Interactionism [14], as these are relevant for this topic area and reflect shared understanding emerging from cross-team discussions.

The second element is a qualitative study, comprising interviews and focus groups, on the mental health needs of and availability of support and services for older Colombians. This will be assessed via semi-structured interviews with health and social care professionals and members of Turbo community associations. A maximum of 10–15 interviews will be conducted, according to thematic saturation. In addition, up to 12 focus groups will be conducted in rural and urban areas to assess older population’s needs, priorities, concerns and preferences. Specifically, there will be four focus groups with older adults, four with older adults’ caregivers, and four with officials and community leaders. This will be supplemented by a stakeholder analysis to construct an inventory of organisations developing and delivering initiatives and services to support elders’ mental health and wellbeing, their resources, focus, capacities and networks. Qualitative transcripts will be translated into English and analysed using thematic analysis by both Colombian and UK research team members. Members of the UK team will attend these focus groups as virtual observers/facilitators. Topic guides will be specifically developed for this study.

The third element is a quantitative survey of a random sample of older adults’ mental health. Older adults will be asked to complete a survey with trained local health-workers and researchers. This includes the WHO-5 Well-being Index [15], Mini International Neuropsychiatric Interview [16], Inventory of extreme experiences in the context of the Colombian armed conflict [17], functional capacity [18], and cognitive impairment [19]. To calculate the sample size, the Epidat 4.2 software was used, opting for a stratified random sampling with proportional allocation according to the place of residence of the older adult. We will survey 446 adults aged 60 + (including an additional 10% for replacement due to losses or design errors). We will use a probability random sampling method stratified by conglomerates with proportional allocation to place of residence. The survey will undergo initial piloting prior to full data collection. Information from medical and administrative records from secondary sources will also be processed. Routine health and other relevant datasets are collected and collated at national state and municipality level. The team already has close working relationships with the data managers and data access at the University of Antioquia. The Antioquia team works closely with the local Turbo Mayor, Municipality health and care community workers and are well placed to negotiate with national and local government organisations, to build systems to track routine data on social and mental health for this project. The relevant data elements will be determined during the early phases of the project, including the systematic review. Data access and quality will be assessed and piloted once the key relevant elements have been determined and combined. The Antioquia team have already reviewed available secondary data sources to support the programme, and are thus familiar with their extent and limitations. Existing data accessible to the team includes SIVIGLA (national public health surveillance system) which includes health service usage and a range of relevant public health datasets within that system; social security and healthcare activity database (SISPRO) through which many relevant datasets can be accessed including data about victims of violence, chronic health conditions and disability, services for elders, social programmes and census data. To characterise the local population, we will use the SISBEN III database, at Municipality level and relevant elements of the national DANE database. These cover a wide range of socioeconomic and demographic factors relating to area populations, including homelessness, substance misuse Quality of Life data, household structures etc. Data will be analysed using SPSS.

#### Part II – Co-producing and developing a community-based mental health intervention

Part II addresses research question 2 and involves three elements: The first element is a systematic review into the effectiveness of community-based psychosocial mental health interventions for older adults residing in LMICs, particularly post-conflict. We will also gather information on implementation barriers and facilitators, where reported. Specifically, we are wanting to understand what interventions have been conducted and how effective these have been and how they have been implemented. The systematic review protocol is registered on PROSPERO [Ref: CRD42021271404]. We will deliver a descriptive evidence synthesis outlining the evidence, applicability and acceptability of psychosocial mental health interventions in relevant settings to inform the third element (co-producing a tailored intervention).

The second element are qualitative interviews with experts in developing and delivering mental health interventions for older adults in LMICs: We will conduct virtual semi-structured interviews linked to the systematic review findings, to help guide the development of the psycho-social intervention, and to understand different potential barriers of implementation in LMICs, with international experts in global post-conflict mental health, including multi-disciplinary academics and clinicians. We will conduct up to 20 virtual interviews, and data will be analysed using thematic analysis. The interview topic guide have been co-developed with public advisors, clinicians and academics, and are newly designed. Interviews will be transcribed and coded by two research team members trained in qualitative data collection and analysis, supported by input from the wider team. This study has received ethical approval from the University of Liverpool prior to data collection [Ref: 10216], with data collection currently ongoing.

The third element is a series of co-production workshops in Colombia with older adults, local government and community organisations, and health care professionals from the Turbo region, joined by the UK team face-to-face and virtually. At the workshops, we will synthesise the findings from Part I and the systematic review and qualitative interviews with experts as part of Phase II to co-produce the mental health intervention via group tasks and discussion. We will conduct a series of three workshops, with up to 20 attendees each. The series of workshops will help refine and co-produce the intervention and produce a brief therapy manual of the intervention, including assessment, problem formulation, therapeutic plan and core components, review of progress, as well as barriers and facilitators to implementation.

#### Part III – Conducting and evaluating a mental health intervention

The planned psychosocial community intervention aims to improve the mental health of older adults in the Turbo region, which will be prepared for implementation across Colombia and other LMICs, and be adapted for relevant UK settings. This proposed solution arose from need expressed by senior staff within the municipal administration of the District of Turbo, then developed jointly between the UK and Colombian team. This endorsement and agreement will facilitate starting this research project swiftly with strong support from local government. It is anticipated to reduce investment costs for the Turbo District Government Plan.

The intervention will be based on existing guidelines for psychosocial interventions, such as Self-help Plus [20] or World Health Organisation’s Mental Health Gap Action programme (mhGAP) Intervention Guide [13]. The precise approach and theoretical framework will emerge based on the findings from Part I and Part II, including the co-production of the intervention with relevant stakeholders in Colombia. Findings from Part I and II will also help in identifying the individual components of the intervention, to also overcome previously experiences barriers in accessing mental health support. This is also the case for the delivery of the intervention, as this may either be delivered individually or in a group setting, all to be decided during co-production. Thus, this psychosocial intervention is very strongly co-produced, ensuring that the needs of older people who are the target of the intervention, as well as their local contexts, are fully taken into consideration to ensure the best possible and suitable intervention for this population.

The effectiveness of the intervention will be evaluated using a mixed-methods approach. We will conduct 15 qualitative semi-structured interviews participants and 10 interviews with community leaders and organisations who have been involved in the set up and delivery of the intervention. We will also quantitatively explore the intervention’s efficacy by collecting baseline and follow-up data after 4 months and 12 months, with the survey described in Part I.

### Co-production and public involvement

This project has been conceptualised with members of the public, and is involving older adults or those caring/having cared for an elderly person in both the UK and Colombia, as well as Colombian community leaders and organisations throughout the project. In particular, the research team has held detailed consultations with the administration of the municipality of Turbo, specifically with the Ministry of Health, and the Mayor’s office who is participating directly in the project by the District Secretary for Health, Protection and Social Welfare and the District's Elderly Program. Older adults are involved in all aspects of this study as public advisers and experts by experience. We have two older adult public advisers in Colombia, and two in the UK to be part of our formal project team. In addition, we have two local government representatives from the Turbo region as professional advisers on the project. Public advisers contribute to all aspects of this project, from designing study documents and designing the intervention, to interpreting findings, reading through study documents, and helping with the dissemination of the findings. Public advisers are reimbursed for each meeting and activity they have carried out, and get their travel expenses paid for.

The group will comprise of between five and eight older adults from various socio-economic backgrounds, and will help shape the study and interpret its findings by providing different real-life experiences of stressful live events. For each meeting, participants will receive a cash reimbursement and their travel expenses reimbursed.

An additional element of co-production and public involvement involves the development of the intervention, the process of which is outlined in Phase II.

### Cross-cultural knowledge exchange

This study benefits from cross-cultural knowledge exchange and learning, which strengthens the development of the community-based mental health intervention, as well as builds capacity in both research teams. Some of the data collection will be conducted in the UK, and the UK is equally involved in the analysis and dissemination of all research, as is Colombia with all aspects of the study. This not only enables methodological skill transfers between both country teams, but site visits in Colombia will also increase cross-cultural learning and understanding the mental health systems in Colombia.

## Discussion

This study protocol provides a manual for co-producing a community-based mental health intervention for older adults in LMICs, which is grounded in evidence base, cross-cultural knowledge exchange, and in-depth co-production and public involvement. Considering the lack of adequate mental health care in LMICs in general, and in Colombia specifically, yet a great need in the community of this post-conflict country [5, 10], this study will provide a novel solution to addressing the mental health needs of the older population by providing a feasible, easily implementable, and affordable intervention.

Given the established links with community groups in the affected region as well as the support from the Ministry of Health in Colombia, the intervention is likely to be continued subsequently. If effective, the intervention can be rolled out across the country, and with the aid this study protocol, can be adapted and implemented in other LMICs also. There are implications for high-income countries however too. Northern Ireland for example has been affected by decades of troubles and political unrest [21], whilst the North West of England is one of the most disadvantaged regions in the country, with higher mortality rates and reduced access to health and social care services than the rest of the population, amplified in the current COVID-19 pandemic [22]. Thus, the intervention can also be adapted by utilising the co-production approach from Phase II. This showcases the cross-cultural knowledge exchange and the findings from this study will have wide implications that reach beyond regional or country lines.

## Data Availability

Not applicable.

## Abbreviation

LMICs: Lower- and middle-income countries
